# Exploring the Tumor Microenvironment in Osteosarcoma: Driver of Resistance and Progression

**DOI:** 10.3390/cancers17193106

**Published:** 2025-09-24

**Authors:** Aidan A. Schmidt, Advay Prasad, Alex R. Huisman, Mark R. Wakefield, Yujiang Fang

**Affiliations:** 1 Department of Microbiology, Immunology & Pathology, Des Moines University, West Des Moines, IA 50266, USA; 2 Department of Surgery, University of Missouri School of Medicine, Columbia, MO 65212, USA; 3 Ellis Fischel Cancer Center, University of Missouri School of Medicine, Columbia, MO 65212, USA

**Keywords:** osteosarcoma, tumor microenvironment, immune cells, immunotherapy

## Abstract

Osteosarcoma (OS) is the most common bone-based cancer in both the US and the world in children, teenagers, and young adults. It is an aggressive form of sarcoma which forms mainly in the long bones of the legs and arms, often metastasizing prior to diagnosis. Every year across the globe, there are approximately 28,000 new cases, yet this sarcoma remains difficult to manage with standard treatments, partly due to its solid and immune resistant tumor microenvironment (TME). The quantity of research conducted on OS because of these difficulties has greatly increased over the past decade, meaning a comprehensive review of new findings on the TME may prove beneficial. This article aims to give a broad overview of the components of the TME of osteosarcoma, discuss its resistances and detrimental effects, and illustrate current and future immune therapy treatments which effectively target the microenvironment. Additionally, it will seek to highlight any knowledge gaps in the current literature and propose further studies to improve clinical outcomes. These studies could be beneficial in increasing drug and treatment efficacy for OS.

## Simple Summary

This review gives a broad summary of the contents of osteosarcomas tumor microenvironment, including immune cells, non-immune cells, extracellular matrix, signaling pathways, and the environment surrounding the tumor. It also discusses some immunotherapy treatments, their shortcomings, and potential solutions to these issues. The goal is to identify areas of weakness in research and highlight where further studies could be effective in creating new therapies to better treat the still poor overall survival rates in osteosarcoma patients.

## 1. Introduction

Osteosarcoma (OS) is a malignant tumor of the bone, most frequently the metaphysis of long bones such as the femur and humerus. While symptoms vary, the site of origin will commonly present with pain, decreased mobility if near a joint, and a palpable mass [1]. OS is typically derived from mesenchymal cells and can become cancerous through many factors, including most commonly genomic alterations in TP53 and Rb [2,3]. The TP53 gene codes for the transcription factor p53, which has effects on DNA repair, cell cycle control, apoptosis, and more, making it an extremely important tumor suppressor gene [4]. Rb is another tumor suppressor gene, the loss of which causes uncontrolled cell proliferation as the G1 restriction point cannot be controlled effectively [5]. A review of OS incidence and survival in the US from 1975 to 2017 found a peak in OS in children and young adults from 10 to 24 years old, with a smaller secondary peak from 80 to 84 years old in male patients. Overall, higher incidences were seen in black and male patients, with females and young cases (0–9) having the highest 5 year relative survival rate when compared to the average of 65.9%, calculated from the last decade of the study with modern treatments [6]. Other studies corroborate this, estimating 70% 5 year survival rates in localized disease, although when OS metastasizes, it drops drastically to a 20–30% 5 year survival rate [7,8,9,10]. This is particularly problematic for OS as it metastasizes early, with approximately 15% of newly diagnosed patients already having cancer spread to the lungs [11]. Treatment for OS typically is focused on a combination of surgery and chemotherapy, potentially adding in radiotherapy for areas that are high risk for surgery, such as the spine. However, OS has relatively high radioresistance, which leads to difficulties in treating these areas [12]. Additionally, therapies that are non-specific for OS have proven to work with low efficacy and have many harmful side effects at effective doses [13]. An important factor in identifying new treatments for OS is the results of transcriptomic studies for highlighting critical genes that may be involved in the progression of the disease. Recent results by Poudel and Kõks display that genes such as IFITIM5, CHTRC1, PANX3, MMP13, TMEM119, and CENPF are upregulated, while hemoglobin and cell checkpoint genes are downregulated to promote tumor survival [14]. These studies and others like them are vital for creating new therapeutic strategies that can effectively target OS.

One of the defining features of OS is its immunosuppressive tumor microenvironment (TME). Generally speaking, the TME of cancer is composed of all the non-cancerous cells in the tumor, the physical components which make up the stroma, and metabolites and signaling molecules. This means that everything from host immune cells and fibroblasts to extracellular matrix (ECM), lymph nodes, and chemokines compose the microenvironment of a tumor [15]. Recent studies suggest that the microbiome of the body can also be an important factor in influencing the TME. The role that bacteria play is unknown, but there is a consistent presence of bacteria in specific cancer types, such as cervical and gastrointestinal cancer [16,17]. In OS, the TME is immunosuppressive, with low infiltration of T cells and noted resistance to many forms of treatment [18]. Immune cells in OS can play both anti-tumor and tumor-promoting effects depending on the cell type, drawing importance to the elucidation of cellular pathways for more effective treatment [19]. The ECM is another important component of OS, a three-dimensional scaffold of proteins and carbohydrates that supports rapid growth and metastasis of the tumor through providing a structure to build on and specific signaling mechanisms [20]. The formed environment of OS is also important in supporting tumorigenesis, with hypoxia stifling healthy cells and increasing angiogenesis and disadvantageous cytokine expression for further growth and potential metastasis [21].

## 2. TME Components

The TME is composed of a variety of different cells and physical components that contribute to the formation and growth of OS. This paper has split this into the categories of immune cells, non-immune cells, and physical components.

### 2.1. Immune Cells

#### 2.1.1. Tumor-Associated Macrophages

Tumor-Associated Macrophages (TAMs) are a subset of macrophages that are believed to originate from circulating monocytes or polarization of resident tissue macrophages due to local signaling and the microenvironment of the tumor [22]. Macrophages can be of two subsets: M1 and M2. M1 are classically activated by toll-like receptors and interferon-γ, and are important for inflammation, while M2 are activated by interleukins (IL) 4 or 13, and are used for anti-inflammatory and healing processes. Because of this, the M2 is generally seen more commonly in tumors and is negatively correlated with survival time and positive clinical outcomes. M2 macrophages are more commonly referred to as TAMs than M1 [23,24]. It is important to note that the classification of M1 and M2 works at a broad level, and macrophage plasticity is not a simple dichotomy, with macrophages showing some ability to adopt a mixed phenotype according to the demand of the local microenvironment [25,26]. TAMs are extremely important in OS, forming up to 50% of the tumor mass and being involved in a variety of pro-tumor processes such as increased tumor angiogenesis, intravasation, growth, and tumor evasion [27].

TAMs support angiogenesis through the secretion of the vascular endothelial growth factor (VEGF), expanding the tumors’ access to oxygen and other nutrients via a transient disassociation of the endothelial cell junctions of the local vasculature [26,28]. This process in the vasculature also contributes to intravasation through tumor microenvironment of metastasis (TMEM) doorways. These doorways are composed of a TAM expressing the Tie2 receptor tyrosine kinase and producing VEGF, an endothelial cell, and a tumor cell expressing the protein Mena. When the VEGF loosens the cell junctions, it allows the tumor cell to slip through the endothelial layer and into the bloodstream [28,29]. This process has been studied in breast cancer, and although no specific research has been conducted into the process in OS, it is possible that similar methods could be present [30]. Furthermore, Tie2 inhibitors such as rebastinib, which reduce TAM activation, have shown promising results in reducing TMEM doorways’ efficacy and metastasis in mouse models of breast and pancreatic cancer, further emphasizing the importance of TAMs to the process of metastasis [31,32]. In OS specifically, Han et al. found that TAMs’ promotion of COX-2 and subsequent phosphorylation of STAT3 lead to increased incidence of metastasis and invasion both in vitro and in animal and patient tissue analysis [33].

TAMs also support the proliferation of cancerous cells in the TME via immunosuppression, specifically to cytotoxic T and natural killer (NK) cells [34]. The first way that TAMs cause immunosuppression is through modification of the PD-1/PD-L1 pathway [35]. In normal physiological states, this pathway stops immune cells from targeting healthy host cells while identifying foreign cells to be destroyed [36]. TAMs produce transforming growth factor β (TGF-β), a cytokine that typically suppresses inflammation and is pro-apoptotic for malignant cells [37]. This cytokine functions to upregulate PD-L1 on the surface of cancer cells in solid tumors, preventing them from being targeted by the immune system [34]. A second method of immunosuppression comes from TAMs’ release of prostaglandin E2 (PGE2), which can upregulate PD-L1 expression through a variety of pathways, such as AKT/mTOR pathway in ovarian cancer, and the COX-2 system in bladder cancer [38,39]. Additionally, PGE2 proved capable of stimulating differentiation of naïve T cells to regulatory T cells (Treg) in vitro and in a mouse lung cancer model by utilizing FoxP3, which will further increase local immunosuppression [40]. Importantly, PGE2 buildup may also transform M1 macrophages to an M2 form, resulting in an increase in the overall number of TAMs at the tumor site and causing a positive feedback loop as they release more PGE2 [41]. A third TAM-produced cytokine deeply involved in OS progression is IL-8, which was shown to promote OS proliferation and metastasis by utilizing the FAK axis in a study by Tatsuno et al. [42]. This research builds upon an earlier 2019 study, which found that circulating OS cells increase production of IL-8 to promote tumor growth, invasion, and metastasis [43].

TAMs support tumor progression through immunosuppression of host immune cells and upregulation of pro-metastasis and invasion factors. Due to their importance in this TME, future research could focus on ways to reprogram these cells or nullify their effects to allow for the greatest effect of standard therapeutic drugs. Identifying specific mechanisms of action for TAM effects will also aid in developing effective treatments. Similarly, as stated above, TAM’s influence on the PD-1/PD-L1 pathway can help underly the mechanisms behind OS progression. Future research can help to further elucidate the unique aspects of an OS’s TME and show potential for novel therapeutics to be developed. Overall, TAM is a crucial aspect of OS and its related TME.

#### 2.1.2. Tumor-Associated Neutrophils

Neutrophils are a short-lived immune cell originally formed from bone marrow hematopoietic cells, which differentiate into granulocyte monocyte progenitors. These undergo further maturation when stimulated by granulocyte colony-stimulating factor (G-CSF), finally being released into the bloodstream when expressing the C-X-C chemokine receptor types 2 and 4 [44,45]. They play an important role in non-specific immune responses to infection and inflammation through crosstalk with immune and non-immune cells, reactive oxygen species (ROS) production, inflammation, neutrophil extracellular trap (NET) formation, and tissue repair [45]. These neutrophils are then activated by the TME to either an N1 anti-tumor or an N2 pro-tumor form. The N1 form works through its high production of ROS and natural cytotoxicity, along with immune crosstalk [46,47,48]. N2 neutrophils are suspected to aid in upregulating angiogenesis and lymphangiogenesis, rebuilding ECM, and producing pro-tumor cytokines such as tumor necrosis factor α (TNF-α), IL-6 and 10, epidermal growth factor, fibroblast growth factor 2 (FGF2), VEGF, and TGF-β [48].

NETs are a unique product of neutrophils consisting of a DNA skeleton, various surface proteins, histones, inflammatory compounds, and neutrophil elastase [46]. Their purpose in a healthy individual is to prevent disease by capturing and eliminating bacteria and other pathogens, but they can also contribute to pathological states such as lupus, periodontitis, atherosclerosis, thrombosis, sepsis, psoriasis, diabetes, post-injury inflammation, and cancer [49]. NETs can be induced by many mediators, such as IL-8, CXCL1 and 2, nitric oxide (NO), cathepsin C, toll-like receptor ligands, G-CSF, platelets, exosomes produced by tumors, and amyloid-β from cancer-associated fibroblasts [49,50,51,52,53,54,55,56,57]. NETs have many important effects on cancer growth. They increase the release of growth factors and proangiogenic factors from proteolysis of the ECM and degranulation of neutrophils, such as VEGF, platelet-derived growth factors, or matrix metalloproteinase 9 (MMP9) [49]. NETs also facilitate the intravasation and metastasis of tumors by dilating the tumor vasculature [58]. Additionally, in a hepatocellular carcinoma model, NET associated with cathepsin C was shown to promote invasive phenotypes [59]. This is further supported by a pancreatic study, which found that NETs promoted migration and invasion by involving the EGFR/ERK pathway [60]. Finally, due to the histones and DNA structure of the NETs, they are able to activate the ILK-β-Parvin system via the CCDC25 pathway, leading to higher levels of cell proliferation, migration, and adhesion in mouse models, suggesting that there is an association with metastasis [61].

Recently, more studies identifying the specific effects of NETs on OS have been completed, with findings generally supporting a similar correlation between NET concentration and OS prognosis. Lin et al. found that a NETScore, consisting of measurements of four specific genes related to NETs, was linked with suppression of T cell function, including proliferation, activation, and migration, along with different drug sensitivity [62]. A 2025 retrospective cohort study provided further insight into the risks associated with NETs, identifying high NET formation with poor response to neoadjuvant chemotherapy and an overall survival 17.8 months lower than those with low NET formation in pediatric OS. Additionally, metastatic sites in these patients displayed elevated NET creation compared to primary tumors [63]. This could potentially suggest that NETs play a similar role in the metastasis of OS as is seen in other forms of solid cancers, although further studies will be required to extrapolate the specific pathways utilized.

Neutrophils and NETs can function in both the initiation and progression of cancer. A 2020 rodent study on lung cancer showed that neutrophils amplified the genotoxicity of urethane due to its ROS production, increasing neoplastic transformation, and a knockout of neutropenic granulocyte colony-stimulating factor led to reduced lung tumorigenesis [64]. Additionally, the production of microRNA (miR-155) by neutrophils may contribute to DNA break errors in repair in colorectal cancer [65]. While it is unclear whether these specific mechanisms are at play in OS, neutrophils play a significant role in OS progression, with studies finding an elevated neutrophil-to-lymphocyte ratio leading to greater resistance to treatment and reduced overall survival [66,67,68]. A 2023 study further found that release of mitochondrial DNA post-surgery made the lungs more vulnerable to formation of a pre-metastatic niche, with an influx of neutrophils allowing circulating OS tumor cells to progress the disease despite effective removal of a primary tumor [69]. A possible pathway for this metastasis is the differentially expressed neutrophil gene PPP2R5C, which utilizes the PI3K/AKT pathway and may be an important marker in future studies for metastasis and recurrence [70].

While the effects of neutrophils in OS have been elucidated, much more research remains to be conducted in order to identify specific pathways that these cells target, as well as the interplay between neutrophils and bone cells in the TME. Future studies should also focus on therapies that could reduce the metastasis-promoting effects of neutrophils and NETs, as patient outcomes are significantly higher for the disease pre-metastasis. In this regard, it is possible that further studies of the PI3K/AKT pathway can help undercover the mechanisms behind OS metastases. In addition, the NET behavior already researched in colorectal and lung cancers is a valuable insight into the potential behavior in OS. Therefore, further exploration into these niche behaviors, specifically in OS NETs, should be taken into consideration.

#### 2.1.3. Myeloid-Derived Suppressor Cells

Myeloid-derived suppressor cells (MDSCs) are a population of immature myeloid cells derived from bone marrow. The main groups of these cells are polymorphonuclear (PMN-MDSCs) and monocytic (M-MDSCs). As can be inferred by their name, these cells play an immunosuppressive role [71]. M-MDSCs are capable of differentiating into PMN-MDSCs when the retinoblastoma gene is silenced, while inflammatory factors can promote their differentiation into macrophages, and hypoxic environments will lead to TAM formation [72]. MDSCs can secrete immunosuppressive substances such as TGF-β, IL-10, and COX2, while promoting tumor growth with substances such as VEGF, MMP-9, and basic fibroblast growth factor [71]. M-MDSCs express high levels of inducible nitric oxide synthetase, leading to the formation of large amounts of NO, which has been shown to inhibit proliferation and induce apoptosis in T cells. In addition, this NO can react with a superoxide anion to form peroxynitrite, which is capable of nitriding the T cell receptor complex, which causes loss of specificity in these immune cells, further increasing immune evasion [73]. This inhibition of T cells is further amplified by the impairment of T cell trafficking by NO, with the molecule decreasing E-selectin expression on tumor vessels, reducing the entry of the T cells [72]. PMN-MDSCs utilize less NO and mainly generate high levels of ROS by upregulating NADPH oxidase and activating STAT3 [71,74]. Interestingly, MDSC in bone metastasis of breast cancer express PD-L1, allowing them to inhibit T cells and promote osteoclastogenesis [75]. While they may not play exactly the same role in OS, there is a distinct possibility of similar processes based on the location of the cancer, which warrants further study.

Beyond T cells, MDSCs also suppress dendritic cells (DC), NK cells, and B cells, although the mechanisms for this are poorly studied in OS. PMN-MDSCs have been reported to block cross-presentation of antigens by DC in rodent models through the transfer of oxidized lipids [76]. The expression of VEGF by MDSCs inhibits DC differentiation, and the production of IL-10 also hinders the maturation of DCs and their creation of IL-12 [71,77]. It has been suggested that NK cells and MDSCs have an inverse relationship, with a variety of strategies being used to downregulate the efficacy of NK cells. Cell-to-cell contact is one major method, with TGF-β from MDSC reducing interferon γ (IFN-γ) production and expression of the NKG2D receptor, leading to overall impairment of function [78,79]. Similarly to DC and T cells, the production of IL-10 by MDSCs also reduces the function of NK cells, as does PGE2, which is generated by the COX2 signaling cascade and further stimulates MDSC generation from monocytes [71,78,79,80]. MDSC-generated NO can also play an important role in reducing NK cell function, including antibody-dependent cellular toxicity and secretion of IFN-γ and TNF-α [81]. Furthermore, indoleamine 2,3-dioxygenase (IDO) release from MDSC reduces expression of the NKG2D, DNAM1, and NCR receptors, greatly decreasing the ability of NK cells to respond to tumor cells [78,82]. Finally, B cells can be inhibited by modulation of IL-7 and STAT5 signaling, as well as downregulation of L-selectin to reduce the number of B cells returning to lymph nodes [83,84]. MDSCs can also modify B cells directly by upregulating PD-L1 on the surface, encouraging differentiation into regulatory B cells, which can further inhibit T cell activity [85]. This process has also been directly seen in the transformation of T cells to regulatory T cells, either due to direct cell-to-cell interaction, secretion of factors such as IL-10 or TGF-β, or by expression of ARG1, IDO, or CD40 [86,87].

In OS, MDSCs expressing high levels of IL-1β can bind to tumor cells to activate the NF-kB pathway to promote progression and migration [88]. They also promote angiogenesis, invasion, and metastasis via the release of signaling factors [89]. Multiple studies have shown that inhibiting the function of MDSCs leads to greater therapeutic response in OS. This was displayed by inhibiting infiltration via IL-18 blockade [90], preventing apoptosis resistance with CXCR4 antagonists [91], and reducing cellular function by blocking PI3K [92].

MDSCs have been recognized as an important immunosuppressive cell for years, and an appropriate amount of research has been conducted to further understand the mechanisms they operate with in cancer. While more studies are needed to determine their exact effects in OS and what drugs or treatments are effective, there are encouraging precedents for improved therapeutic benefit in OS when MDSC are targeted. Future research should aim to build off of these studies while also determining which functions in other cancers carry over to OS. The relationship between MDSCs and the TME of OS could hold valuable insights into the pathophysiology of OS.

#### 2.1.4. Mast Cells

Mast cells are bone marrow-derived cells that classically respond to immunoglobulin E-mediated allergic reactions but are also associated with various innate and adaptive immune responses towards pathogens [93]. In the OS TME, mast cell abundance has a negative correlation with prognosis, and a 2021 study further found a correlation between patient death and mast cell infiltration [94]. This may be due to the fact that OS may stimulate mast cells, causing local and systemic inflammation with immune cell recruitment, as well as modulating bone remodeling, potentially through activation of the receptor activator NFκB ligand, which stimulates bone resorption [95].

Mast cells play a variety of roles in cancer, but these have generally been determined in non-OS cancer lines. First, mast cells can stimulate angiogenesis through the release of factors such as VEGF, FGF-2, IL-8, heparin, and TGF-β [96,97,98]. Second, mast cells can induce cancer cell proliferation. Release of IL-6 can induce STAT3 expression, driving growth, IL-17A production increased proliferation while inhibiting apoptosis in a gastric model, and exosomes can have effects on other cell pathways [98,99,100]. Third, mast cells can enhance the process of invasion by releasing IL-8 and MMP-9, stimulating the epithelial–mesenchymal transition by a phosphorylation cascade, as seen in vitro for thyroid, breast, bladder, and non-small cell lung carcinomas [101,102,103,104,105]. Fourth, mast cells can also attract and activate a number of immunosuppressive cells to the TME, such as MDSC, Treg, and macrophages. Finally, mast cells can suppress anti-tumor immunity via secretions such as adenosine, FOXP3, and TGF-β, the last two also resulting in conversion of T cells to Treg. It is important to note that while mast cells do have many pro-tumor functions, they can also act in an anti-tumor manner, recruiting leukotrienes and NK cells, inducing apoptosis, phagocytosing tumor cells, and inhibiting tumor proliferation [98]. Interestingly, based upon their location at the tumor-bone border, mast cells may contribute to osteolysis in OS [106]. In studies of other bone-related disorders, such as osteoporosis and osteoarthritis, mast cells have been found to increase osteoclastogenesis and potentially play a role in healing and angiogenesis in bone fractures [106,107,108]. It is currently unclear whether these functions are preserved in the OS TME or if they operate differently.

While it is clear that mast cells can play an important role in a multitude of cancers, very little research has been conducted on their effects in OS thus far. The previous studies may be used as guidelines or expectations for mast cells, but continued research on these cells will be required to determine their effects in OS and suitable therapeutic targets. Specifically, there is more to learn about how mast cells can help contribute to the metastatic behavior of OS. Research into these fields can help provide support to future pharmaceuticals and develop better outcomes for patients afflicted with OS.

#### 2.1.5. T Cells

**Figure 1:**
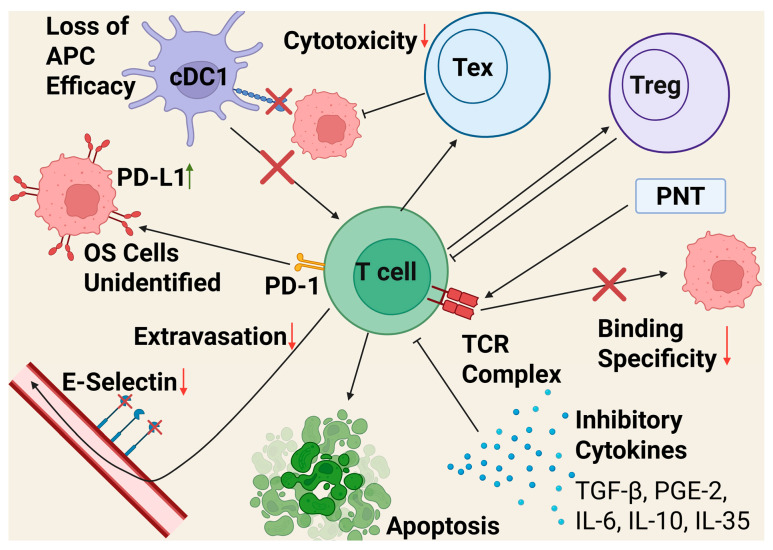
A representation of the variety of ways in which T cells lose their effectiveness in the TME of OS, showcasing the challenges that modern treatments may face when attempting to target only one pro-tumor factor [35,40,48,72,98,111,112,113,114,116,117,118]. Figure created using Biorender.com. (https://app.biorender.com/illustrations/688a7775732c095685a963aa).

T cells are thymus-derived lymphocytes that play a role in adaptive immune responses, both in cellular and humoral immunity. They can differentiate into a variety of forms, including helper T cells, cytotoxic T lymphocytes, and Treg [18]. Figure 1 details the variety of ways T cells are suppressed in the OS TME. In OS, exhausted T cells (Tex) are a major component of the TME, and this seems to be more emphasized in recurrent and metastatic lesions [109]. T cells become exhausted by prolonged antigen stimulation over a period of weeks to months, meaning that their formation is accelerated as a cancer progresses and the body fights against it over time [110]. Tex cells have reduced cytotoxicity against cancer cells compared to standard T cells, and express elevated levels of inhibitory molecules such as PD-1, CD-38, and TOX, with enough epigenetic changes to be considered distinct from effector T cells [111,112,113,114]. Tex cells have a unique relationship with TAMs, with Tex actively recruiting monocytes, which will become TAMs in the TME, while TAMs prime unexhausted T cells for exhaustion, a process that is exacerbated in hypoxic conditions like those found in TME. A 2022 study in mouse models of melanoma and breast cancer found that Tex stimulates this migration and differentiation through CSF1, CCL3, CCL4, and CCL5, while TAMs upregulate exhaustion through antigen-specific binding to the T cell receptors [113]. Further studies have found that Tex cells can play an immunosuppressive role similar to Treg by using CD39, induced by hypoxia, to generate adenosine [115]. In short, T cells that infiltrate the TME rapidly become exhausted over time and lose their efficacy, then further contribute to immunosuppression.

Beyond Tex cells, γδ T cells are a minority of T cells in the body, as most T cells possess an αβ lineage, but they are worth mentioning as they have been correlated with immunosuppression of αβ T cells through PD-1/PD-L1 signaling [119,120]. However, they are also a promising target for future immunotherapy research due to their independence from major histocompatibility complexes (MHC) [121].

Treg cells are a much more researched cell type, with an important role in reducing inflammation and autoimmune conditions. In cancer, Tregs play an immunosuppressive role, expressing CTLA-4, CD25, and FoxP3 and resulting in decreased T cell activity. Specifically, CTLA-4 denies the CD28 of T cells access to CD80/86 on antigen-presenting cells (APC), depriving the T cells of co-stimulation and causing them to be inactivated. CD25, expressed on the surface of Treg, sequesters IL-2 from the surroundings, preventing the molecule from upregulating T cell differentiation. FoxP3 aids this process by inhibiting IL-2 transcription and promoting CD25 expression [116]. GPR15 is another modulator of Tregs, which is significantly correlated with increased FoxP3 and regulates their migration as a chemoattractant [122]. Although this process has not been specifically identified in OS, the supporting data in the lungs may make it an attractive target for future research. Additionally, Tregs secrete molecules including IL-10, IL-35, and TGF-β, which suppress immune function in the TME [116]. The levels of infiltrated Treg in a tumor have been correlated to poorer prognoses and survival rates in multiple cancer types, including lung, ovarian, gastric, breast, pancreatic, head and neck squamous cell carcinomas, and melanoma [123]. While Tregs are vital for protecting the body from its own immune system, they become a key component of tumor defenses when they infiltrate the TME.

T cells can be considered important factors in the development of a TME. While the influence T cells have on OS specifically is still being discovered, their prevalence in the pathogenesis of other cancers showcases a promising field to be explored in OS. Therapies to reduce or slow Tex formation and reprogram Treg may show great promise in OS treatment.

#### 2.1.6. B Cells

B cells are bone marrow-derived lymphocytes that play a crucial role in the adaptive immune system, creating specific antibodies to target various pathogens of the body. This makes them useful in identifying and destroying cancerous cells, but they can also promote tumor proliferation through the secretion of immunosuppressive cytokines and cellular mediators [124]. Similarly to T cells, there exists a class of B cells known as regulatory B cells (Breg), comprising less than 10% of the population in normal physiological states. The cells secrete IL-10 or IL-35, TGF-β, and other regulatory molecules based upon tumor type, reducing T cell proliferation, activity, and secretions. These cytokines also lead to the development of more Treg, which have a strong immunosuppressive effect in the TME [124,125]. Bregs may also express membrane regulatory molecules such as Fas ligand (FasL), which induces apoptosis in effector T cells, to further increase immune evasion [117].

Beyond immunosuppressive effects, some B cells have functions that directly promote tumor growth, as was found in a 2020 study, which discovered a new subtype of B cells that secreted proangiogenic cytokines, including VEGF, cysteine-rich angiogenic inducer 61, andromedullin, FGF2, platelet-derived growth factor subunit A, and midkine [126]. However, research on the effect of B cells in OS is sparse and can be contradictory, with some studies finding B cell infiltration linked to OS progression and poorer prognosis, and others displaying improved treatment results [127,128,129].

The immunoglobulins secreted by B cells can also have varied effects in cancers. While the immunoglobulin G antibody subclass created by B cells is an effective fixer of complement on cancer cells, the immunoglobulin A (IgA) and immunoglobulin E (IgE) subclasses have been correlated with poor prognosis in hepatocellular carcinoma, melanoma, KRAS-mutant lung carcinoma, prostate, and bladder cancers [130,131,132,133,134]. IgA expresses PD-L1 and IL-10 without fixing complement or aiding in antibody-dependent cellular cytotoxicity (ADCC), while IgE specifically was significantly associated with osteosarcoma development in a study by Zhang et al. [135,136]. The reason for this association is unclear and will require further study. To summarize, B cells are a double-edged sword in immunotherapy and should be researched in depth for future treatments, particularly concerning IgA and IgE, due to their history of correlation with cancers. Specifically, interventions that use different B cell subsets can prove useful in understanding the TME. These studies can provide evidence behind treatments and factors that influence the progression of the TME. In terms of OS, there is less research conducted when compared to other cancers, and therefore, it can benefit from the effort of future researchers.

#### 2.1.7. Natural Killer Cells

NK cells are innate lymphoid cells that can exterminate pathogens through the release of cytotoxic granules or the expression of death ligands, as well as generate some inflammatory cytokines such as IFN-γ and TNF-α [19]. Few studies have been conducted on the effects and actions of NK cells in OS, but they have been extensively reviewed in the literature for other cancers, which are likely to show similar mechanisms. In the TME, NK cells are massively suppressed by the quantity of TGF-β present, as this cytokine downregulates expression of the NKG2D and CD16 receptors, thus reducing perforin release and IFN-γ production. This effect on NKG2D is further enhanced by TGF-β downregulates expression of the NKG2D ligand on tumor cells [137,138]. A further 2017 study found that TGF-β can convert NK cells to type 1 innate lymphoid cells, which cannot control tumor growth and metastasis [139]. Other cellular modulators in the TME that inhibit NK function include adenosine, PGE2, IDO, NO, and IL-10, while cells involved in the suppression include TAMs, TANs, MDSCs, Treg, Breg, mast cells, cancer-associated fibroblasts, and endothelial cells [34,78,98,116,124,138]. Generally speaking, these signals can be counterbalanced by stimulatory cytokines such as IL-2, IL-15, IL-18, IL-21, and IL-27. However, an interesting finding from a 2019 study showed that IL-18 actually strengthened the effects of TGF-β on NK cells, reducing expression of Cx3CR1 and NKp30 receptors while upregulating CXCR4. This significantly affected the NK cells’ ability to lyse pathogens [140]. In contrast, the other tested stimulatory cytokines did not amplify all of the effects of TGF-β, but IL-2, IL-15, and IL-21 had either no counter or synergistic effects to the reduction in CX3CR1, and all the cytokines were unable to modulate the increase in CXCR4 [140]. Future studies may benefit from focusing on how specific cytokines act in TME compared to standard physiological conditions.

NK cells can contribute to the immunosuppressive TME in more ways than simply a loss of their typical function. They can differentiate into an angiogenic form, secreting VEGF, angiogenin, and MMP9 while upregulating expression of hypoxia-related genes [141,142,143]. Additionally, tumor-associated NK cells stimulate endothelial cells to express CXCL8, ICAM-1, and VCAM-1 and showed an ability to recruit monocytes while polarizing them towards TAMs [144]. With a 2021 study finding that most NK cells in OS strongly expressed the genes for IFN-γ [109], it seems likely that this vascular growth-promoting transition is not commonly seen, as the angiogenic NK cells have limited production of IFN-γ. Regardless, further research into the subject could provide an advantage for future treatments.

NK cells in the OS TME generally function similarly to their physiological state but suffer from reduced efficacy and may contribute to angiogenesis and recruitment of immunosuppressive cells. Further investigation into treatments that may remove these inhibitions could be valuable in increasing the body’s innate immune response. Similarly, further inquiry into NK cells’ immunosuppressive effects in OS patients can provide crucial details about their cell interactions. Research regarding stimulatory cytokines and their effects on the TME/NK cells would resolve these inquiries and provide a more comprehensive understanding of OS.

#### 2.1.8. Dendritic Cells

DCs are antigen-presenting cells derived from the bone marrow that play a crucial role in activating T cells. A specific class of DCs is known as mature regulatory DCs (mregDCs). A study by Liu et al. discovered that these mregDCs were found in elevated levels in OS compared to bone marrow and were rare in normal peripheral blood, suggesting tumor specificity. These cells express CCR7, CCL17, CCL19, and CCL22, allowing them to recruit Treg, with higher scores of mregDCs equating to poorer survival rates and a positive correlation between mregDC and Treg infiltration. This interaction is mediated through CD274-PDCD1 and PVR-TIGIT signaling [145]. The CCR7 receptor has been found to aid DCs in migration to lymphoid organs and modulate the formation of associated lymphoid tissues in past studies [146] and thus may also have a role in the metastasis of OS from its TME. The TME can also have a reprogramming effect on type 1 conventional DC (cDC1), with elevated levels of PGE2 from TAMs leading to the loss of transcription factor IRF8. Due to this dysfunction, the cDC1s were unable to activate effector T cell responses with the same efficacy, greatly undermining the immune response to the tumor in melanoma [118].

Beyond their effects on T cell infiltration, DCs contribute to tumor growth through the loss of the glutamate metabotropic receptor 4 (GRM4). In human models, GRM4 acts as a tumor suppressor, and its loss leads to accelerated OS tumor development through IL-23 elevation and IL-12 reduction. High levels of IL-23 were found to correlate with worse overall survival in these models [147]. While little research has been conducted on its exact effects in OS, IL23 is known for being a proinflammatory cytokine that can become involved with multiple pro-tumor processes, particularly in breast cancer, where it has been correlated with higher tumor size and stages [148]. These include recruiting M2 macrophages and neutrophils to increase secretions of immunosuppressive TGF-β and IL-10, stimulate expression of VEGF for angiogenesis, and activating NF-κB signaling to cause further immune cell activation and tumor growth [149,150]. Further study on IL-23 suppressing drugs or GRM4 agonists in OS could be a valuable area for future research, particularly due to the potent T cell activating abilities DCs possess in their normal physiological state and the high levels of T cell suppression found in the OS TME.

### 2.2. Non-Immune Cells

#### 2.2.1. Osteoclasts

Osteoclasts are cells responsible for bone resorption in human beings, with their proliferation linked to the development of OS. Growth and cell formation of osteoclasts are heavily governed by the RANKL/RANK signaling pathway, where the nuclear factor kappa-B ligand (RANKL) stimulates bone resorption [151]. Osteoclasts will ultimately differentiate from hematopoietic stem cells, but there are occasions where they form the differentiation of tumor-associated macrophages [151]. OS presentation is often associated with the dysfunction of osteoclasts, where their upregulation helps facilitate tumor cell proliferation. It has been recognized that OS cells use small extracellular vesicles (sEVs) to communicate with their TME. One example involves miR-19a-3p, which, when packaged into an sEV, promotes osteoclastogenesis. This specific microRNA will target the phosphatase tension homolog/phosphatidylinositol 3 kinase/protein kinase B signaling pathway, which is known to increase bone resorption and tumor-induced osteolysis in mouse models [152]. This interaction highlights the dependence of tumor biology on bone resorption, suggesting that the OS cells can directly influence osteoclast development. The presence of a positive feedback loop has also been involved in osteolytic OS. Osteoclastogenesis mainly depends on the RANK/RANKL pathway, and OS tumor cells can potentially produce a surplus of RANKL [153]. This imbalance will often lead to a disruption of normal bone remodeling due to an increase in osteoclast resorption. Bone resorption naturally secretes byproducts such as growth factor TGF-B. TGF-B will, in turn, support OS development and RANKL production, establishing a positive feedback loop between osteoclasts and tumor development [154]. Beyond their role in bone remodeling, osteoclasts help to influence the TME through their interactions with other stromal components. In conjunction with the RANKL, sialic acid–binding immunoglobulin-like lectin (Siglec-15) can be used by osteoclasts to ultimately stimulate the RANK/RANKL pathway [155]. In OS patients, Siglec-15 will contribute to the characteristic positive feedback cycle. Siglec-15 has another important role where it acts as an immune regulator, downregulating T cell proliferation and supporting the TME [156]. These findings demonstrate that osteoclasts play a crucial role in the development of the TME, thereby supporting the crosstalk between bone resorption and tumor immune escape [157].

The RANKL signaling system also plays a role in the relationship between osteoclasts and immune cells. Specifically, helper T cells and regulatory T cells will secrete RANKL to regulate bone resorption in the microenvironment. Similarly, RANKL can also stimulate dendritic cells to promote the proliferation of T cells [158]. The interaction between immune cells and the RANKL signaling system highlights potential future areas of study in how they influence the development of OS. Also noteworthy is an immunoglobulin-like receptor known as OSCAR. OSCAR works alongside RANKL to stimulate bone resorption and osteoclast development from stem cells. OSCAR dysfunctions have also been implicated in immune disorders such as rheumatoid arthritis [159]. While it is unclear whether these systems influence OS, they are promising areas of study regarding OS and the TME [159]. Similarly, developing therapeutic modalities to impact the RANKL system could provide valuable insights into the progression of OS and provide new evidence regarding its TME.

#### 2.2.2. Osteoblasts

**Figure 2:**
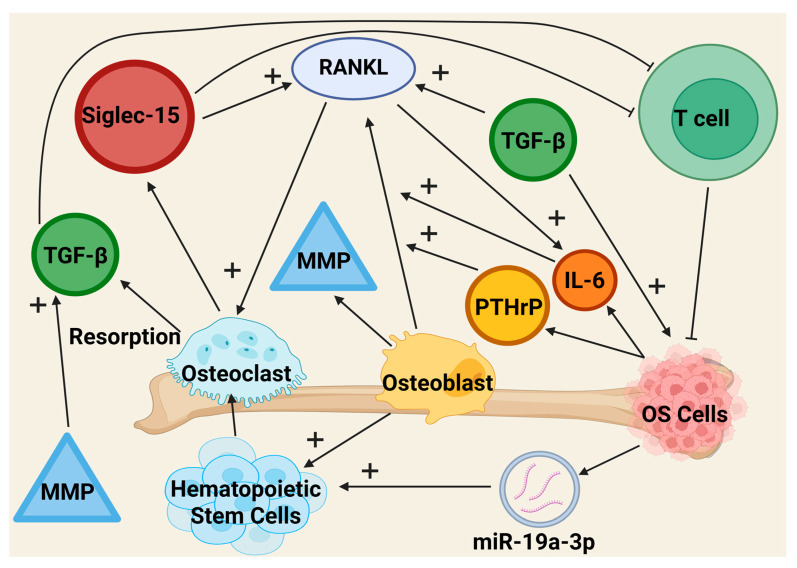
A schematic detailing some of the intricate interplay between osteoclasts, osteoblasts, and OS cells, displaying some of the vicious cycles that lead to the aggressive nature of the cancer. Figure created using Biorender.com (https://app.biorender.com/illustrations/6889509d56b22489b5d7c7b8).

Osteoblasts are critical cells involved in bone remodeling with three main functions. First, they are the primary cells responsible for bone formation and the regulation of bone remodeling. These tasks are not just limited to skeletal structure but extend to the surrounding ECM. Secondly, through the use of RANKL, osteoblasts direct the production of osteoclasts and, as mentioned before, direct osteoclast activity and differentiation. Lastly, osteoblasts assist hematopoietic stem cells’ renewal by releasing osteopontin, thrombopoietin, and angiopoietin-1 [160]. Osteoblasts are derived from mesenchymal stem cells (MSCs), and their cell cycle/differentiation is influenced by the RUNX family transcription factor 2 (RUNX2). Osteoblasts also produce proteinases and MMPs, which are signaling factors osteoclasts use to promote bone remodeling; however, these MMPs can also influence malignancy within OS by stimulating TGF-β production, promoting tumor cell survival [154]. RUNX2 is critical for proper osteoblast differentiation and bone production, but has also been linked to OS occurrence [160]. RUNX2 has both tumor suppressor and oncogenic properties, making its expression dependent on the environment. Although much is left to be studied, overexpression of RUNX2 leads to proteins that assist in tumor formation [154,161]. It is also important to note the lineage of an OS multifaceted with evidence showcasing both an osteoblastic and MSC origin [162]. OS tumor cells have also been observed to release signaling factors such as parathyroid hormone-related protein (PTHrP) and IL-6. Increases in these factors will upregulate the production of RANKL by osteoblastic cells [163]. This phenomenon is similar to the positive feedback loop mentioned before regarding osteoclasts. RANKL production will increase in osteoclastic activity, which accelerates the production of tumor-supporting factors. The interplay between osteoclasts, osteoblasts, and OS cells is summarized further in Figure 2. Overall, supporting pro-tumorigenic osteoblast activity and perpetuating the positive feedback loop characteristic of OS.

Osteoblasts’ interactions with the host immune system also play a role in the development of OS. Specifically, this cell line is involved in lymphopoiesis, helping regulate the development of T cells and B cells. For B cell development, osteoblasts will support the commitment of B cells from HSCs through the interactions of VCAM1 between cell types, IL-7, and CXCL12 [164]. T cells have shown the ability to influence the growth and development of both osteoblasts and osteocytes through interactions with parathyroid hormone (PTH). Generally, T cells will provide the cell signals that influence the development of osteoblasts from MSCs, specifically priming these osteoblasts to become more sensitive to PTH and targeting the activity of mature osteoblasts using TNF-α and IL-17 [165]. Further study of OS specific interactions with the immune system is needed to draw further conclusions. However, these results between osteoblasts and immune cells can provide the foundation for future studies of OS.

#### 2.2.3. Fibroblasts

Fibroblasts fall under non-immune cells with various functions, ranging from extracellular matrix remodeling, angiogenesis, and wound healing. However, tumor cells can manipulate fibroblasts by secreting IL-1, IL-6, TNF, and TGF-β, which have proinflammatory functions [166]. In the TME, fibroblasts that become subjected to periods of inflammation or fibrosis are termed cancer-associated fibroblasts (CAFs). CAF is a broad term describing various subtypes of fibroblasts and is phenotypically different than typical fibroblasts. Therefore, a CAF’s function can be varied, enabling a pro- or anti-tumorigenic response in the TME. [167] In OS studies, CAFs engaged in a more pro-tumorigenic response by increasing the proliferative abilities of tumor cells. The modification of tumor cell behavior by CAFs are due to the fibroblasts’ use of exosome-mediated intercellular communication. [168] These exosomes often contain microRNA (miRNA) that can impact various oncogenic pathways of OS. While many of these miRNAs have yet to be studied, a well-studied example is miR-1228. MiR-1228 has a direct suppressor of cancer cell invasion, or SCAI, a tumor suppressor protein commonly found in breast cancer tumors. Its downregulation has a direct influence on the development of both breast cancers and OS [169,170]. Overall, CAF-mediated exosome transfers provide a promising new frontier in the study of osteosarcomas, with evidence to support a connection between CAFs and the development of the TME and, overall, the migration of OS.

### 2.3. Extracellular Matrix

The extracellular matrix (ECM) has been linked to developments within the TME [20]. Multiple components of the ECM can contribute to the development of OS. Some of the more studied subtypes include collagen types I, III, fibronectin, laminin, and proteoglycans. All five of these subtypes within the ECM of OS are upregulated and have various outcomes on the progression of OS [171]. Collagen I, for example, can upregulate matrix metallopeptidase 2 (MMP-2), which can promote OS metastatic capabilities [20]. Regarding OS, the surrounding matrix has shown a higher mechanical rigidity when compared to healthy cells. OS tumor cells cultured on a hydrogel, used to mimic bone stiffness, exhibited enhanced survival, traction forces, and the formation of cancer stem-like populations identified by CD44/CD133 markers. These phenotypes were not observed on softer substrates, more typical of non-bone tissues [172]. The high matrix stiffness has the potential to promote epithelial-to-mesenchymal transition (EMT) and cell migration in OS. EMT is the attainment of mesenchymal-like qualities in epithelial cells, causing cells to lose their polarity and cell adhesion properties, adopting a more malignant/invasive behavior [173]. In OS, EMT will mediate the nuclear translocation of myosin-related transcription factor A (MRTF-A), a transcription factor involved in EMT gene expression [174]. Currently, the interplay between these two factors has not been explored completely. However, some studies have shown that ECM stiffness upregulates MRTF-A expression in certain cases, which increases EMT and the progression of OS development [175]. Together, these findings demonstrate that ECM stiffness helps direct OS advancement by promoting cell migration, EMT, and cancer stemness through transcriptional reprogramming. ECM stiffness can induce biochemical drug resistance in various tumor cell lines. Cells that undergo EMT have an increased number of ABC drug transporter proteins. It is also important to note that increased ECM stiffness negatively affects ABC drug transporters, where greater stiffness will result in a less effective pump [176]. ABC transporters actively efflux chemotherapeutic agents, often reducing treatment efficacy [177].

EMT is linked to OS development through its connection with MRTF-A. Therapies targeted at the MRTF-A system have the potential to influence OS development and should be considered for future areas of study. The ECM should not be underemphasized when discussing OS. The multiple facets of ECM etiology, such as matrix stiffness, MMP-2, EMT, etc., all contribute to OS pathophysiology. Further identification of ECM components will provide a deeper understanding of OS and can elucidate further treatments related to OS.

### 2.4. Signaling Pathways

The progression and proliferation of osteosarcoma are accompanied by observable changes in the signaling pathways responsible for evading apoptosis, promoting growth, and facilitating the progression of osteosarcoma through the activation of mesenchymal stem cells [178]. These pathways provide valuable insight into the impact that OS has on the microenvironment around it, as well as provide potential targets for therapeutic intervention.

#### 2.4.1. PI3K/AKT/mTOR Signaling Pathway and Osteosarcoma

The phosphatidylinositol 3-kinase/protein kinase B/mammalian target of Rapamycin (PI3K/Akt/mTOR) signaling pathway functions broadly to regulate cell mobility, growth, proliferation, adhesion, and survival. Immunostaining analysis in primary OS cases has shown that the pathway is abnormally expressed and that the upregulation is associated with poor prognosis with OS [178]. Additionally, the path is subject to frequent change, which contributes to the proliferation and metastasis of OS. One source of this genetic variation is the PIK3CA gene, which acts through both lipid-like and protein kinase activity to contribute to an upregulation of AKT activation, contributing to a reduction in apoptosis and increased tumor infiltration in OS cell lines [179].

Moreover, the upregulation of the PI3K/AKT/mTOR pathway has been associated with alterations in the chemosensitivity of OS [180]. Doxorubicin, a member of the anthracycline family, is a chemotherapeutic agent commonly utilized in high-dose regimens paired with methotrexate and cisplatin in the treatment of OS [181]. In OS-derived chemotherapeutic studies, doxorubicin provides valuable insight into the interaction between chemoresistance and the PIK3/Akt/mTOR pathway. In a kinome-wide CRISPR screen, Zhang et al. identified an amplification of PRKDC, a gene whose downstream effects amplify the phosphorylation and activation of Akt, as a critical determinant of DOX resistance in OS. When mouse xenografts and human organoid models were treated with the PRKDC inhibitor AZD7648 and DOX, tumor suppression was improved [182]. In another study, He et al. illustrated that the administration of DCC-2036 (an inhibitor of the HCK / AKT / mTORC1 axis) promoted autophagy in OS tissues [183]. Together, these findings underscore the therapeutic relevance of the PI3K/AKT/mTOR axis in OS and highlight its potential as a multifaceted target for overcoming chemoresistance. Also, the potential influences of the non-immune and immune cells discussed in this paper on the PIK3/Akt/mTOR should be further studied. These can provide valuable knowledge into how OS communicate with the TME and how the disease progresses in patient populations.

#### 2.4.2. Notch/Hedgehog/Wnt/120 and Osteosarcoma

The Notch/Hedgehog/Wnt/β-catenin pathway represents a complex, interconnected system of ligands, receptors, coreceptors, and nuclear transcription factors whose interplay is broadly responsible for the initiation and regulation of cell differentiation, stem cell self-renewal, growth, cell cycle progression, and proliferation [184]. Multiple studies support that increased activation of this pathway is common in OS and is associated with tumor progression, greater lung metastasis, and a worse prognosis [179,185].

In a study conducted by Jiang et al., osteosarcoma tissues harvested from 48 patients exhibited an upregulation of Wnt-promoting miRNA, FLVCR1-AS1, relative to controls. This regulator’s level was also found to be higher in stage III OS in comparison to stages I-II, suggesting the pathway’s role in OS progression [186]. In another study conducted by Chang et al., inactivation of the Wnt/β-catenin pathways with polyphyllin I (PPI) reduced OS viability, migration, and growth, while increasing apoptosis and cell cycle arrest when treated with tumor necrosis factor-related apoptosis ligand (TRAIL) [187].

Beyond growth and metastasis, this pathway has been implicated in the ability of osteosarcoma to autoregulate cancer stem cells (CSCs) and metastasize [179]. Using c-FOS-driven OS models, Matsuoka et al. illustrated that selective deletion of the Wnt-less (Wls) gene led to reduced tumor number, volume, lower proliferation and mineralization, and the production of fibroblast-rich and less aggressive tumor phenotype in comparison to control samples [187]. These studies suggest the importance of this axis in the progression of OS as well as its importance as a potential therapeutic target in the future. The Notch/Hedgehog/Wnt/β-catenin pathway has been explored for its relation to the TME in numerous studies, but in regard to OS, there is still more to be explored. This pathway is a critical step in the formation of cancer cells and should be further studied in the context of OS, as well as identifying suitable targets for treatments.

### 2.5. Environment

Through its influence on signaling cascades and homeostatic mechanisms, osteosarcoma exerts a profound effect on the bone microenvironment around it. This phenomenon functions to propagate the progression and metastasis of OS as the shift in environmental characteristics fundamentally changes the behavior and interactions of OS and the cells around it [176,188]. An understanding of this influence on the bone microenvironment is necessary to see the systemic effects of this cancer, as well as to provide potential therapeutic targets to slow or halt the progression of OS.

Hypoxic conditions are a common hallmark of many cancers. As tumors grow beyond homeostatic boundaries, they exert a demand for oxygen and resources that exceed the capacity of local venous networks to supply them [189]. In response to this, tumors will adapt and aberrantly express hypoxia-inducible factor 1α (HIF-1α) to generate their own blood supply [178]. Liu et al. illustrated the necessity of HIF-1α in the progression of OS as upregulation of the inhibitory miRNA, miR-20b, downregulated the expression of HIF-1α and suppressed the tumor line’s invasive status and proliferation rate through suppression of the VEGF pathway [190]. The generation of a hypoxic microenvironment also contributes to the high necrotic rate exerted by OS, as consumption of resources reduces the oxygen available below physiological levels [191]. This hypoxic microenvironment changes the behavior of local and tumorigenic cells. As OS continues to grow beyond the blood supply and undergo necrosis, its cells shift their primary energy source from oxidative phosphorylation to fermentation, resulting in an overproduction of lactate that causes the local pH to drop [192]. In addition to providing a physiological niche for the tumor to grow, the acidification of the microenvironment promotes OS progression through the activation of mesenchymal stem cells. Short-term acidic stimulation exerts an increased expression of stress factors, growth factors, and immunoregulatory molecules that enhance the ability of OS to grow and metastasize [178]. All of these moves collectively create an environment both harmful for the tissues around OS as well as beneficial for the unique metabolic capabilities of the tumor. Interventional methods that impact this environment represent a valuable clinical opportunity that warrants further attention. The expansion of miRNA treatment research, as seen in studies like Liu et al.’s, may be a necessary step towards developing safer and more effective therapeutic agents against OS.

## 3. Immunotherapy Treatment

Current treatment of OS typically involves neoadjuvant chemotherapy followed by surgical excision of the tumor. Common drugs include doxorubicin and methotrexate [193]. However, progress in outcomes has seen little improvement over the past decade due to the immunosuppressive nature of the TME as well as the aggression that OS exhibits. Due to this, immunotherapy is increasingly becoming a heavily researched field for the treatment of OS, along with ways to counteract the TME to ensure maximal benefit of the treatments.

Immunotherapy involves modulating the body’s own immune system to fight against cancer, an excellent option for OS due to the rampant immune evasion ongoing in the TME. Immune checkpoint inhibitors (ICI) are a promising set of medicines that target proteins that regulate immune activity, such as CTLA-4, PD-1, TIM-3, and LAG-3 [194]. These proteins are found on T cells and stop T cells from eliminating other cells by binding specific ligands on the other cell. The most common inhibitor for CTLA-4 is the drug ipilimumab, while there are many PD-1 inhibitors, including nivolumab, pembrolizumab, avelumab, durvalumab, camrelizumab, atezolizumab, and more [195]. Alone, most of these drugs have mixed results in OS, with ipilimumab showing no notable tumor regression in a phase I trial from 2016 [196], while four trials of PD-1 inhibitors from 2017 to 2020 had similarly underwhelming results [197,198,199,200]. Despite this, when used in conjunction, the two drug classes showed some results, with a 2022 study showing 6 of 41 patients having sustained partial responses or stable disease [201]. There are multiple possible reasons for the limited effectiveness, including limited T cell infiltration into the TME of OS or T cell inactivation by way of other immune cells. Regardless, other ICIs have been identified, and trials have begun. TIM-3 inhibitors, such as sabatolimab, have been used with PD-1 inhibitors in solid tumors. Results have been similar to combinations of PD-1 and CTLA-4 inhibitors, with limited responses to treatment [202,203]. Relatlimab, a LAG-3 inhibitor, was approved for use in metastatic melanoma in conjunction with nivolumab in 2022 as Opdualag due to multiple successful studies [204,205,206]. However, few studies have been conducted on the effects of LAG-3 inhibitors on solid tumors such as OS. The first-in-human phase I study of dual LAG-3 and PD-1 inhibition in advanced solid tumors was conducted by Mao et al. in 2024 and found response rates of 75% for non-small cell lung carcinoma and 70.6% for HER-2-gastric cancer [207]. While ICIs remain a promising topic for future research, there needs to be more of a focus on their effects in OS, particularly in combination therapies, due to the complex and interwoven nature of the TME.

One way to directly address the TME would be therapies and drugs specifically targeted towards the immunosuppressive cells of the TME. A benefit of this approach is that it would allow ICIs and other commonly used therapeutic drugs to have a greater effect, as the drugs targeting the TME could help reactivate the immune system’s defenses or reduce the amount of inhibition. Table 1 details the various components of the TME with potential therapeutic implications. TAMs can be combated in a variety of ways. A 2021 study focused on reprogramming TAMs from the M2 form to an M1 anti-tumor form and did so by utilizing M1 exosomes, which targeted M2 IL-4 receptors while carrying small interfering RNA and microRNA to cause M1 polarization. This inhibited tumor growth in mouse models of breast and lung tumors [208]. The TAMs’ function may also be reduced by elimination by zoledronate or other agents [209], inhibiting recruitment through silencing of CCL2/CCR2 or CSF/CSFR signaling [22,210], or reinstatement of phagocytic abilities via release of CD47 and CD24 antibodies [211,212]. Additionally, COX-2 blockers such as aspirin and celecoxib have been shown to have positive effects in reducing metastasis and invasion in OS, providing another pathway to reduce TAM effects [33].

DCs are another potential target that could be targeted by vaccine therapy. An older 2015 study of DC vaccines utilized in neuroblastomas and sarcomas found 2 of the 10 patients to have cleared the disease, but the small sample size makes further research necessary [215]. A more recent 2024 study on OS in lung metastases found that a CD103+ cDC1-based vaccine caused tumor regression and enhanced infiltration of T cells into both treated and untreated tumors, indicating a systemic response [213]. This is an especially important finding when considering the fact that metastatic disease is already commonplace when OS is first diagnosed [11]. These DC vaccines have also been tested alongside other checkpoint inhibitors, as was shown by Zhou et al. in a phase I study, where more frequent injections of the DC vaccine lead to a disease control rate of 90% as opposed to 44.4%, displaying the positive effect that the combination of multiple immune therapies can cause in soft tissue sarcomas and OS [214].

MDSCs have a strong inhibitory effect on anti-tumor immunity, which has led to a large number of studies focused on reducing their presence in the TME. Common chemotherapeutic drugs such as cisplatin, doxorubicin, and ifosfamide can eliminate these cells, a trait shared by all-trans retinoic acid in breast cancer models [216,217]. This can also be achieved by limiting CSF-1, as this is a major stimulant for MDSC production. A potential drug for this condition undergoing clinical trials is pexidartinib, which showed decreased tumor growth and prolonged time to metastasis in mouse models of OS [218]. Unfortunately, data for this drug seems to be somewhat unclear, with phase II trials on solid tumors finding little efficacy, while a phase III trial on tenosynovial giant cell tumors found that it had a significantly higher overall response rate than the placebo. Conversely, a 2024 study loaded pexidartinib into M1 macrophages for delivery and found a significant improvement to treatment results in OS and tenosynovial giant cell tumors [219,220,221,222]. It is unclear whether the difference in delivery is responsible for this gap in outcome, but direct elimination may be a viable method to improve patient outcomes. Beyond these treatments, MDSC-focused treatments may target the CXCR4 receptor, which can be bound by an antagonist such as AMD3100 to decrease tumor growth and increase T cell infiltration. This effect was especially pronounced in conjunction with a PD-1 inhibitor [91,223]. In addition to these methods, IDO inhibitors, such as epacadostat, have been shown to be effective in reactivating anti-tumor immunity and inhibiting tumor growth in OS [224]. As is the case with many of the new immunotherapy drugs, more research needs to be conducted in OS models, as current data is limited.

Chimeric antigen receptor T cell (CAR-T) therapy is another innovative immunotherapy approach that isolates patient T cells from blood, modifying them to express specific receptors, and then culturing them before reintroducing the cells to the host. Second-generation models of CAR-T add a co-stimulatory domain, which makes them more durable and effective, with the majority of current therapies being in this category [225]. This therapy has shown great promise in hematological malignancies, but research is still in progress for solid tumors such as OS [226]. Many challenges hinder the use of CAR-T in OS, including the immunosuppressive TME, low specificity, and a lack of identified targets, limited durability, and significant side effects [225]. Multiple studies have been conducted to solve these issues. New cellular targets for CAR-T therapy include ALPL-1, which is highly and specifically expressed in both the primary OS tumor and metastatic lesions [227]. Identification of OS targets like this helps to solve a major complication of CAR-T cell therapy in OS. CAR-T co-expression of CXCR5 and IL-7 has also been shown to decrease expression of PD-1, TIM-3, and TIGIT while increasing Bcl-2 expression and T cell infiltration, enhancing eradication of OS tumor cells and survivability [228]. This novel method of promoting T cell effect has excellent potential for future uses in solid tumors. Durability concerns have been addressed in a variety of ways, but an intriguing one is the combination with PD-L1 inhibitors, which was effective in enhancing tumor control and survival in mouse OS models [229]. CAR-T cell therapy has seen great advances in efficacy for treating OS, and further advancements, particularly in combination therapy, may deliver excellent results.

## 4. TME Evolution with Disease Progression

The components of the TME have been discussed, but it is a dynamic environment that can drastically change in composition based on disease progression. Early forms of OS feature greater immune cell infiltration, demonstrating the body’s initial resistance to the tumor. Yang et al. showed that when compared to metastatic lesions, non-metastatic OS tumors had higher immune and stromal scores, with greater amounts of NK cells, immature B cells, M1 macrophages, and neutrophils. In contrast, M2 macrophages decreased compared to metastatic tumors [230]. While this was not specifically demonstrated in early versus advanced primary tumors, it is likely that early tumors would display stronger immune resistance. A 2022 study lends credence to this thought, identifying that genes for unfavorable prognoses included those that lead to neutrophil degranulation, osteoclast differentiation, and upregulation of the fibroblastic network, potentially linking CAFs to the advancement of disease [231]. Indeed, CAF involvement in OS progression appears to be quite common, although the specific effects are not always as clear. Zhihao et al. found advanced OS tumors often have greater quantities of CAF [232], a finding supported by Hu et al. in their 2024 study, which also remarked on the increase in collagen found in advanced tumors [233]. Logically, this makes sense, as recruitment of more CAFs will lead to a more pronounced effect on the ECM. As has been previously discussed, ECM in OS is stiffer than baseline forms [172], allowing for MRTF-A upregulation and a higher metastatic potential [20,175]. Beyond CAF and ECM increases, decreases in T cell infiltration at advanced stages have been noted [233]. In both early and late stages of OS in non-metastatic lesions, the PI3K-Akt signaling pathway has been identified as an important driver of progression [234]. It is abnormally upregulated throughout OS cells and may aid in evading apoptosis and increasing tumor cell infiltration [178,179].

Metastatic lesions share many similarities with primary lesions, but there are key differences. As the metastatic tumor forms, it creates a phenotype of chronic wound healing in the surrounding tissues. This induces fibrosis and leads to an increase in macrophage and epithelial intermediate infiltrates [235]. The roles of osteoclasts and osteoblasts also undergo a change in metastatic lesions. Osteoclast infiltration is significantly reduced compared to primary lesions, while osteoblasts transdifferentiate into malignant chondroblastic cells [109]. These chondroblasts may contribute to the ECM richness seen in metastatic lesions, similar to advanced OS lesions, with the density of the surrounding matrix [233]. While the PI3K-Akt pathway remains an important process in these lesions, the MAPK pathway is more dominant in pulmonary metastatic lesions, potentially lending itself to being a target for future treatments [234].

The greatest issue with identifying the composition of the OS TME at different stages is the lack of longitudinal studies to track progression. Cross-sectional studies allow for some patterns to be identified, but it would be greatly beneficial to have continuous data from multiple patients to see the changes over time. In the same vein, longitudinal studies with immunotherapy treatment could aid in discovering exactly how these drugs affect the composition of the cells in the tumor. Multiple challenges will need to be faced when acquiring this data, chief among them the rapid metastatic potential of OS, which causes 15% of patients receiving a diagnosis to already have metastasis [11]. Beyond this, the age range of patients with OS may cause difficulties in standardizing a progression timeline due to the difference in immune response between children and young adults. However, if this information were to be collected, it would allow for more specific targeting of immune cells based upon the stage of the disease, potentially leading to better overall survival, making it a worthwhile field of study for future research.

## 5. Future Perspectives

The current state of OS research provides many potential avenues for the development of safer and more targeted alternatives to conventional chemotherapies. Future directions of treatment for OS could go in many directions due to the multifaceted resistance displayed by the TME. Immune cells and their role in the TME are a well-documented phenomenon that would yield further understanding if studied more extensively in the context of OS. Macrophages, which form the bulk of the immune cell mass in the OS TME, are a prime target for further study [27]. Their influence on the PD-1/PD-L1 pathway is a massive driver of drug resistance to checkpoint inhibitors, and their ability to create a positive feedback loop utilizing PGE2 secretions and Tex cells may give resistance to most standard treatments [35,41,113]. Additionally, TAMs have been shown to be key players in metastasis of OS, suggesting that this could be an ideal target to prevent the spread of the tumor from the primary lesion [33]. Neutrophils represent another area that could improve our understanding of the pathogenesis of OS. While neutrophils contribute to the initiation and progression of lung cancer, models that specifically study their impact on OS could enhance understanding of the pathogenesis of the disease as well as provide future therapeutic targets [65]. For example, a study conducted by Lin et al. found that high NET formation led to poor response to neoadjuvant chemotherapy, suggesting a potential axis to combat chemotherapy-resistant OS [62]. Additionally, the role of myeloid-derived suppressor cells as an immunosuppressant in the TME could be further explored in the context of OS to determine their exact role in the drug resistance of OS specifically [71,72,73]. The role of lymphocytes in the TME of OS is another area that would benefit from further study. Tex, and their prevalence in the TME of recurrent metastatic lesions of OS, could provide a potential target for the understanding of metastasis in OS and possibly illuminate opportunities to prevent it [109]. While B cells were found in a 2020 study to contribute to the production of proangiogenic cytokines, their specific effect on OS remains unclear [127,128,129]. Further understanding of these cells in the context of OS may enhance knowledge of disease progression and provide new areas to target therapeutically. Natural killer cells play a unique, almost bimodal, role in the progression and prevention of OS, as their suppression allows for the immune evasion characteristic of OS, and their transition into an angiogenic form may assist the progression of OS [137,138,139,141,142,143]. However, current understanding of NK and its role in the TME is limited and would massively benefit from more extensive, OS specific clinical research.

In addition to immune cells, non-immune cells also represent an exciting opportunity in the progression of understanding and treatment of OS. Osteoclasts and their role in the proliferation of OS, as well as their status as the axis between immune cells and non-immune cells through RANKL signaling, are essential targets for therapeutic consideration in the future [151,153,157]. Further understanding of this axis and the process that converts de novo osteoclasts into tumor-associated osteoclasts would provide invaluable insight into the progression of OS and potential measures to intervene. Additionally, osteoblasts and their similar modification of the RANKL pathway and their role in the overproduction of TGF-β represent a similar area that deserves more attention [154,160,161].

Beyond further research regarding the TME, critical analysis and expansion of current therapeutic modalities are necessary for the improvement of OS-related therapeutic outcomes. The recent results of Zhou et al. are very encouraging, displaying the effectiveness of DC vaccines when utilized with immune checkpoint inhibitors in controlling OS progression [214]. The systemic effects of vaccines also would make this a field for further study, as it enables resistance to lesions that may be impractical or high-risk for surgery [213]. Finally, the most important field of research will likely be combining OS immunotherapy treatments to find the greatest results. Multiple studies have shown that the highly resistant TME in this cancer displays better prognoses when affected by a combination of treatments rather than monotherapy [196,197,198,199,200,201,202,203]. Although substantial progress has been made in understanding OS, a comprehensive characterization of each component of the TME remains critical for the identification of effective therapeutic targets and for advancing the overall understanding of the disease. Future studies should focus more on identifying cellular targets throughout the progression of the disease to attain maximal efficacy for patient treatment. Furthermore, clinical trials emphasize the combination of immunotherapies with activation and suppression of various immune cells to determine the most useful combinations will be vital.

## 6. Conclusions

This review has highlighted the complex nature of the tumor microenvironment in osteosarcoma, including the immune cells, non-immune cells, extracellular matrix, signaling molecules, and local factors. It has also discussed emerging treatments in immunotherapy and possible methods to counteract the immunosuppressive environment that these drugs must function in, and highlighted some of the gaps in the current literature to reveal where further research is needed to improve patient prognoses. Of particular importance is identifying which medications can work well together in a synergistic manner, as results have largely been poorer than expected when used as a monotherapy. However, the complexity of the TME allows for many angles to approach the problem, giving hope for further studies and clinical trials to find an ideal mix of therapeutic options to improve survival outcomes in patients with osteosarcoma.

## Figures and Tables

**Table 1:** TME cell function in OS and therapeutic implications.

TME Cell	OS Carcinogenic Function	Therapeutic Implications	Ref.
TAM	Promotes angiogenesis through increased VEGF, immunosuppression via PD-1, increased incidence of metastasis and invasion through promotion of Cox-2 and IL-8	ICI less effective if used as monotherapy, treatment that reduces function correlated with better prognoses	[22,28,33,35,42,210,211,212]
Neutrophil	NET formation, causing T cell suppression with a potential role in metastasis; when elevated causes an increase in drug resistance; aids in formation of pre-metastatic niche	Development of drugs targeting NETs could be massively beneficial, reducing infiltration may increase treatment efficacy	[62,63,66,67,68,69]
MDSC	Immunosuppression through secretion of TGF-β, IL-10, and COX2, promotion of tumor growth via VEGF, MMP-9, and FGF, promotes angiogenesis, invasion, and metastasis, progression and migration via NF-kB pathway	Improvement noted when MDSC function inhibited; targeting of secreted factors may improve therapeutic results	[71,88,89,90,91,92]
Mast Cell	Infiltration correlated with death, stimulates bone resorption, local and systemic inflammation	Reducing infiltration and activation potentially beneficial	[94,95]
T Cells	Immunosuppression through Tex and Treg	Tex are major components of OS TME, drugs targeting them or increasing their anti-tumor properties may be effective	[109,116]
B Cells	Infiltration potentially linked to poorer prognoses, secretion of IgE correlated with OS development	IgE blockers may reduce inflammation and OS development	[127,128,129,135,136]
NK Cells	Impaired cytotoxic effects allow for further tumor growth, potential weak angiogenic contribution	Further research required, reestablishing NK cytotoxicity potentially advantageous in immunity	[109,137,138,144]
DC	Recruit Tregs, potential role in metastasis, tumor growth through loss of GRM4 and elevation of IL-23	DC vaccines shown to be useful in treated and untreated lesions and beneficial with ICI	[140,146,147,213,214]
Osteoclasts	Dysfunction correlated with tumor cell proliferation, TGF-β release supports OS development and RANKL feedback loop, downregulates T cell proliferation via Siglec-15	RANKL feedback loop disruption may be a valuable target for decreasing bone remodeling	[152,154,156]
Osteoblasts	Stimulation of TGF-β production requires RUNX2, which is linked with OS occurrence, upregulate production of RANKL	RUNX2 inhibitors may reduce OS growth	[154,161,162]
CAF	Increases proliferative abilities of tumor cells through exosome-mediated intercellular communication	Reducing TME inflammation may decrease CAF formation; targeting exosomes may be helpful	[166,168,169,170]
ECM	Upregulation of MMP-2 promotes metastasis, increased mechanical rigidity enhances survival, EMT, and migration, and inhibit ABC transporters	Drugs that utilize ABC transporters may be ineffective; treatments targeting MMP-2 may prove beneficial	[20,172,175,177]
